# Advanced Cellular Models for Preclinical Drug Testing: From 2D Cultures to Organ-on-a-Chip Technology

**DOI:** 10.3390/cancers14153692

**Published:** 2022-07-28

**Authors:** Valentina Foglizzo, Emiliano Cocco, Serena Marchiò

**Affiliations:** 1 Department of Biochemistry and Molecular Biology, University of Miami Miller School of Medicine, Miami, FL 33136, USA; vxf221@med.miami.edu (V.F.); exc2752@med.miami.edu (E.C.); 2 Department of Oncology, University of Torino, 10060 Candiolo, Italy; 3 Candiolo Cancer Institute, FPO-IRCCS, 10060 Candiolo, Italy

**Keywords:** cancer, target therapy, 3D tumor models, organ-on-a-chip, nanotechnology

## Abstract

**Simple Summary:**

Novel strategies that aim at personalizing cancer therapy are in rapid evolution. In the past decade, new methods to test for the efficacy of either standard-of-care medicines or novel targeted compounds have been implemented. In this review, we introduce the reader to experimental studies that employ patient-derived material to produce spheroids, organoids, or organs-on-a-chip as platforms that allow a more accurate representation of cancer complexity compared to bidimensional cell cultures. We discuss on the versatility and reliability of these model systems, provide evidence of their usage in drug screenings, and describe potential downfalls. The open question is whether or not tumor mimicry in vitro will be, in the near future, advanced enough to prospectively inform about treatment outcome on a certain patient.

**Abstract:**

Cancer is a complex disease arising from a homeostatic imbalance of cell-intrinsic and microenvironment-related mechanisms. A multimodal approach to treat cancer that includes surgery, chemotherapy, and radiation therapy often fails in achieving tumor remission and produces unbearable side effects including secondary malignancies. Novel strategies have been implemented in the past decades in order to replace conventional chemotherapeutics with targeted, less toxic drugs. Up to now, scientists have relied on results achieved in animal research before proceeding to clinical trials. However, the high failure rate of targeted drugs in early phase trials leaves no doubt about the inadequacy of those models. In compliance with the need of reducing, and possibly replacing, animal research, studies have been conducted in vitro with advanced cellular models that more and more mimic the tumor in vivo. We will here review those methods that allow for the 3D reconstitution of the tumor and its microenvironment and the implementation of the organ-on-a-chip technology to study minimal organ units in disease progression. We will make specific reference to the usability of these systems as predictive cancer models and report on recent applications in high-throughput screenings of innovative and targeted drug compounds.

## 1. Introduction

Cancer treatment requires the effort of different professionalities. A multimodal approach usually combines surgical treatment for disease sampling, removal radiotherapy to reduce the need for invasive surgical procedures, and drug administration to kill cancer cells [1]. Classic chemotherapeutic agents with cytotoxic properties are still widely used in the clinic. However, as these drugs affect essential processes and are not able to discriminate between diseased and healthy cells, they often produce important on-mechanism side effects and organ failures [2]. This is the reason why, also based on the massive sequencing effort to identify novel molecular targets and/or driver genes, more specific drugs have been produced in the past few decades. Most of these drugs are small-molecule compounds that still inhibit essential proteins; as a consequence, they display narrow therapeutic windows that need to be carefully evaluated in terms of dose-response and biodistribution between the tumor and the healthy tissues.

The overall cost of a drug entering the market is estimated in the range of billions of dollars. Although targeted therapeutics have opened up new roads in cancer therapy, the number of new chemical entities receiving FDA approval is constantly decreasing [3]. Furthermore, it has been shown that only 5% of those entities prove to be efficacious in clinical trials [4,5]. The reasons why not many drugs enter the clinic, in spite of promising preliminary results, reside in the fact that preclinical experiments do not translate well to the human. This has to be attributed to the type of experimental models we are still using.

Bidimensional (2D) cell cultures have been and are still widely employed in high-throughput drug screenings, despite the high degree of false negative/positive hits due to the poor recapitulation of the in vivo complexity, and particularly the lack of cell–matrix interactions and the absence of an apicobasal polarity. Tridimensional (3D) cell models, such as spheroids, recapitulate some features of a tumor’ spatial architecture, yet they do not include fundamental components such as the tumor vasculature and microenvironment. Animal models, where most of the drugs are tested preclinically for their efficacy and pharmacological properties, often return an imperfect readout because of species-specific differences. Furthermore, most of the animal models used in research do not have a complete immune system. Should we not forget that animal research is costly, time consuming, and ethically questionable. New technologies are being implemented to optimize in vitro tumor models as an efficient surrogate of animal studies.

In this review we will focus on how the in vitro model systems panorama is changing under the impulse of fulfilling the need to envision more successful clinical trials using tools that show a high degree of flexibility, a controlled environment, and that are amenable to high throughput screenings. We will discuss 3D model systems and the organ-on-a-chip technology, making specific reference to those experiments that employ primary human cells and tissue specimens for the evaluation of anticancer drug activity.

## 2. Bidimensional Cultures of Cell Lines: Traditional Cancer Models

Cancer cell lines remain the major source of biological material for the screening of active agents in cancer therapy. Cell lines have the capacity to proliferate indefinitely and to be propagated for long periods of time. They are amenable for high throughput screenings, and panels of cell lines with defined mutations are available for controlled in vitro experiments, such as the ones from the National Cancer Institute (last updated in 2015) [6]. Cancer genomic data coupled with pharmacologic profiles have shown to be good predictors of drug sensitivity in the Cancer Cell Line Encyclopedia, a collection of 947 human cancer cell lines [7]. Furthermore, the ease with which it is possible to manipulate immortalized cancer cells with genome editing systems such as CRISPR-Cas9 makes them an invaluable tool to study gene function and drug sensitivity [8,9]. Lastly, we are now able to generate drug-resistant cell lines to investigate the molecular mechanisms (both genetic and epigenetic) that render a certain type of tumor refractory to treatment [10,11]. Cell lines, however, have intrinsic limitations. First, they have to be adapted to specific culture conditions and this favors clone selection thus impinging on cell heterogeneity. Second, it is not always easy to generate cancer cell lines, being prostate cancer a prominent example with only seven cell lines established. In addition, their ability to proliferate indefinitely comes at the expenses of cell differentiation; indeed, progressive changes in gene expression profiles have been documented [12]. As an example, the genomic characterization of 27 strains of the breast cancer cell line MCF7 showed substantial genetic variation, probably due to passaging and experimental manipulation. These alterations were reported to have a tremendous impact on drug susceptibility [13].

Patient-derived primary cells represent a more suitable resource for genetic and drug screenings. They better represent the tumor in vivo, although their lifespan is limited to few passages. When cultured in 2D, the contact with the plastic dish makes them change morphology and lose some of the original functions. In addition, the success rate of primary cell lines establishment is quite low and extensive biochemical, histological, and genomic characterizations must be conducted to verify the fidelity of the model [14].

More in general, cell lines do not recapitulate certain in vivo conditions such as peculiar cell–cell and cell–matrix interactions. There is no tumor stroma involved and, as a consequence, cell lines lose the peculiar signals coming from their niches. Furthermore, cells in 2D are constantly exposed to high levels of nutrients and oxygen, parameters that, in vivo, are subject to variability imposed by the tumor architecture [15,16]. It is with no surprise that in this scenario many drugs work in a culture dish but ultimately fail in the clinic. In view of designing more accurate and successful clinical trials, superior preclinical models that incorporate elements of cancer complexity are being developed.

## 3. Tridimensional Cell Cultures: New Models in Cancer Research

### 3.1. Multicellular Tumor Spheroids and Tumorshperes

**Figure 1:**
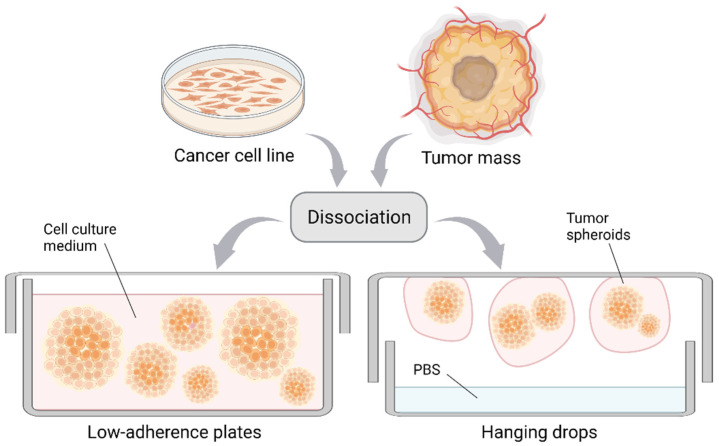
Formation of cancer cell spheroids and tumorspheres. Spheroids are formed from the dissociation of cancer cell monolayers/tumor masses upon cell growth in suspension. This can be achieved by culture in low-adherence plates (**left**) or by applying the hanging drop method (**right**). Created with BioRender.com.

Multicellular tumor spheroids (MCTS) have been successfully established from a variety of cancer cell lines [17]. Indeed, low density single-cell suspensions form MCTS in conventional media when cultured in low-adherence plates or with the hanging drop method (Figure 1). Despite little resemblance to the primary cancer, MCTS display features such as metabolic and proliferative rates similar to the in vivo situation, even though it has been demonstrated that specific culture media are necessary for the complete recapitulation of a tumor in vivo [18]. Compared with 2D cell lines, the MCTS hypoxic core better mimics the gradients of oxygen and nutrients observed in a tumor [19]. Furthermore, this method allows oriented cell–cell contacts, which are fundamental for signal activation between neighboring cells [20,21]. There are multiple protocols to generate MCTS and these are extensively reviewed in Gunti et al., 2021 [22].

Cultivation of spheroids can be scaled up by means of rotary systems that—in their simplest form—consist in a bottle and an agitator. In this way, cells are grown in suspension and high amounts of spheroids can be produced. However, large quantities of media are required and mechanical damage may occur [23]. Rotating wall vessel bioreactors can obviate to the high shear stress imposed by these agitators, being formed by a liquid-filled cylinder that, by rotating on its axis, gently drags the fluid in a circular motion. With this method, spheroids with a more uniform size, increased metabolic activity, and higher oxygenation can be produced [24].

MCTS have also been established from a number of tumor types [25,26,27,28,29,30] and are usually referred to as tumorspheres. For example, colonspheres derived from colon cancer specimens have been shown to share a good degree of similarity in gene expression profile with the original tumor, to retain the ability to form metastasis upon kidney capsule transplantation, and to respond to irinotecan and 5-FU therapies as the corresponding patient-derived xenografts [31]. Tumorsphere formation is considered a readout of tumor stemness [32,33]. It has been demonstrated that these cultures are enriched in stem cells, or tumor-initiating cells, and their serial propagation is a direct reflection of their ability to proliferate and self-renew over time. Cancer stem cells are linked to drug resistance and, in fact, MTCS are believed to more faithfully mirror the response to therapy compared with 2D cell lines [34]. An elegant work by Seguin et al. shows that αvβ3 integrin-positive cells sorted from lung and pancreatic tumors have the ability not only to form spheres but also to replenish the αvβ3 integrin-negative subpopulation and thus to recapitulate the original tumor heterogeneity in vitro. Furthermore, this population is shown to drive resistance to the epidermal growth factor receptor (EGFR) inhibitor erlotinib through the formation of an αvβ3 integrin—KRAS-2B—RalB complex [35]. Resistance to therapy has also been investigated by Gencoglu et al. utilizing a panel of ascites-derived primary ovarian cancer samples. With the purpose of comparing response to therapy in 2D and 3D cultures, they have been able to show that tumorspheres recapitulate cisplatin resistance as seen in vivo whereas 2D cultures do not [36]. Similarly, patient-derived hepatocellular carcinoma cell lines grown as spheres better represent sensitivity to routinely used chemotherapeutics compared to either 2D cell lines or co-cultures [37]. One aspect of therapy resistance is the multidrug resistant phenotype, which is characterized by the presence of specific efflux pumps that counterbalance drug uptake. Azharuddin and colleagues have shown that tumorspheres from patients treated with chemotherapy display differential activity of these pumps and exhibit resistance to therapy, a feature that is not maintained in the 2D cell lines [38].

Spheroids are more and more considered as preclinical systems to test drug activity and to predict therapy response in vitro. In a first example, 117 specimens of endometrial cancer have been employed to produce cancer tissue-originated spheroids (CTOS) with a good success rate (62% of them could be cultured for more than 2 weeks). Two CTOS lines, one of grade 3 adenocarcinoma (cp22), and one of serous adenocarcinoma (cp43) were tested for their sensitivity to 79 molecularly targeted drugs, giving different positive hits. For instance, cp22 was resistant and cp43 was sensitive to Y155A (a survivin inhibitor), and this feature was retained when the spheroids were implanted in nude mice. The efficacy of Y155A was tested on a total of 11 CTOS lines, reflecting a broad range of sensitivity to this drug. Similarly, sensitivity to everolimus (a rapamycin complex 1 inhibitor) was variable, in support of the differential effects observed with patients in clinical trials [39]. In another example, glioblastoma patient-derived cells have been successfully cultivated as spheroids and subjected to high-throughput screening with a library of 1912 drug compounds, which resulted in MEK and PI3K inhibitors to be selected as positive hits. In vivo validation resulted in successful treatment with MEK inhibitor alone as compared with in vitro, where the combination of the two agents had a synergistic role [40]. As a further example, it has been shown in colorectal cancer specimens that paired and spatially distinct spheroid cultures would be better suited to address intra-tumor heterogeneity, and thus therapy response, in vitro [41].

These results show that we are on a stage where tumorspheres are appropriate for high-throughput screenings and can inform on which compounds should proceed to further preclinical investigation. However versatile, this tool can only be considered as an intermediate representation of cancer complexity, as it is based on random aggregation of cells rather than on the recapitulation of the actual tumor architecture. Furthermore, spheroids lack vasculature and matrix-imposed stiffness, which convey survival/death signals.

### 3.2. Patient-Derived Organoids

**Figure 2:**
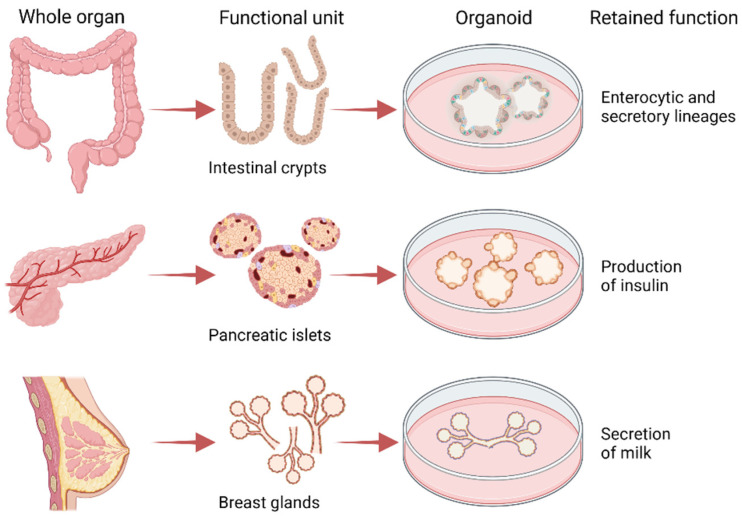
Organoids recapitulate in vitro the architecture and functions of the tissue in vivo. From isolated intestinal crypts it is possible to obtain organoids with enterocytes that absorb nutrients and secretory cells (e.g., Goblet cells) that secrete mucus. From isolated pancreatic islet it is possible to derive organoids that secrete insulin and from isolated mammary glands organoids that secrete milk upon stimulation with prolactin. Created with BioRender.com.

The advent of the organoid technology, which dates back to 2009 [42], has made it possible to propagate a variety of stem cells (adult, fetal, embryonic, induced pluripotent) from which heterogeneous cell populations are derived to recreate tissue architecture and functions in a dish [43]. Specific culture conditions are of fundamental importance for the maintenance of in vivo traits: this is the case of intestinal organoids that in niche-inspired medium preserve both the enterocytic and secretory lineages as they are in the crypts [44], but also of pancreatic and breast organoids that in specific media produce insulin and milk, respectively [45,46] (Figure 2). Media setup is crucial to allow cell proliferation and the onset of lineage-specific gradients that promote differentiation. In other cases, a specific medium—frequently including inhibitors—has to be prepared for differentiation to occur [47]. We suggest the review from Kim et al. for an accurate glimpse of what the media for different type of organoids are made of [48].

As an outcome of this discovery, methods to establish patient-derived tumor organoids (PDTOs) have been developed that can be cultured and expanded in clinically relevant timeframes in the search for personalized therapies [49,50,51,52,53]. Research on PDTOs dates back to one decade ago, when original work on pancreatic cancer has shown the setup of culture conditions for primary pancreatic ductal adenocarcinoma (PDAC) organoids that retained in vitro the same tissue architecture and cell differentiation of the tumor in vivo. The genetic and epigenetic landscape of these tumor varies widely and the lack of drugs targeting the most common mutated genes (KRAS, TP53, CDKN2A, SMAD4) made the authors to select two epigenetic drugs A366 (inhibitors of G9a) and UNC1999 (EZH2 inhibitor) to be used alone or in combination with gemcitabine, the standard-of-care treatment for this tumor. While no growth inhibition was shown in the A366 treated organoids, four organoids out of five tested showed response to UNC1999 and positivity for H3K27me3 markers for which EZH2 is a writer, highlighting the importance of patient-derived cultures in the search of new therapeutic strategies [54].

Genomic studies alone have been shown to be highly informative but rarely effective in identifying useful therapeutic strategies. In search for a valuable pipeline that would ultimately lead to the design of more accurate clinical trials, in 2016 Pauli and colleagues published a paper in which they prepared organoids selected from 145 specimens and performed personalized drug screenings coupled with genomic analysis for four late-stage cancer patients. A uterine carcinosarcoma, an endometrial carcinosarcoma, and two stage IV colorectal cancers with respective mutations on KRAS and APC, were subjected to drug dose-response screenings with a total of 160 drugs. For each tumor, targeted agents were found, which were tested on matched patient-derived xenografts for efficacy and toxicity. Furthermore, for each patient, combinations of targeted agents were identified that were more effective than the standard-of-care [55].

Cancer specimens frequently harbor hundreds of mutations. It is thus quite complicated to assess the relative contribution of targeted therapeutics in a multiple-mutation background. Organoids are amenable for genetic manipulation and isogenic organoids are becoming an invaluable tool for the screening of targeted compounds. It is with this purpose that KRAS G12D organoids have been created from wild-type colorectal cancer specimens to study the response to EGFR-RAS-ERK pathway inhibition, which is ineffective in RAS-mutated cancers. Despite proven on-target toxicity, low concentrations of navitoclax, a BCL-family member inhibitor, seemed to hold great promise in combination therapy by priming mutant RAS tumors for apoptosis [56]. This shows how informative organoid screenings are, and how important would it be to routinely implement them in the preclinical stage before a drug can be tested in vivo.

Pancreatic cancer is one of the deadliest tumors worldwide. Patients sometimes undergo surgery but not all of them are eligible for such treatment, which offers nowadays just a modest improvement to the overall survival. Furthermore, pancreatic cancer is often found as an advanced disease at diagnosis, so treatment options are dismal. Tiriac and colleagues successfully established 114 PDTOs out of 159 samples that recapitulated in vitro the genetic alterations found in the primary tumors. They performed therapeutic profiling (pharmacotyping) of 66 PDAC-confirmed PDTOs, finding fluctuating sensitivity between patient samples to the most commonly employed chemotherapeutic agents (gemcitabine, paclitaxel, irinotecan, 5-FU, and oxaliplatin). They were able to stratify patients into sensitivity subgroups and to confirm their findings in a retrospective analysis of patient response to the chemotherapeutic drugs. Furthermore, they employed targeted drugs as alternative strategies for chemotherapy-refractory PDTOs [57]. Seminal work by Driehuis et al. substantially confirmed these findings by matching the clinical history of the patients and the response to chemotherapeutic drugs of the PDTOs in culture [58]. Biliary tract carcinoma organoids have also been established by Saito and colleagues in order to find novel therapeutic strategies for this often-inoperable disease. By screening 339 clinically and non-clinically used drugs, they have been able to show that the organoids respond to commonly used chemotherapeutics, but also to amorolfine and fenticonazole, antifungal drugs that could be repurposed for cancer therapy [59]. The organoid technology aids in developing innovative therapeutic regimens where, for example, cell lines are not available. This is the case of castration-resistant neuroendocrine prostate cancer (CRPC-NE), an advanced disease derived from CRPC tumors that have lost dependency on androgen receptor (AR) as a way to elude AR-targeted therapy. These resistant tumors have almost no representation in terms of cell lines. This is the reason why Puca et al. generated 4 PDTOs from CRPC-NE tumors and screened 129 chemotherapeutics on them, identifying alisertinib (aurora kinase inhibitor) and GSK343 (EZH2 inhibitor) as novel potential therapies [60].

Organoids can be generated from fine needle biopsies with high efficiency with the purpose of biobanking. Storage of samples that maintain the genetic heterogeneity of a tumor and derivation of samples that represent different tumor histology is an invaluable tool in precision medicine. In 2019, Kim and coworkers established a biobank of 80 lung cancer organoids (LCOs) and matched normal airway tissues with the aim of screening for different drug activity. The LCOs encompassed five histological subtypes (adenocarcinoma, squamous cell carcinoma, squamous adenocarcinoma, large cell carcinoma, and small cell carcinoma) and generated tumors when implanted into immunocompromised mice. Treatment with different drugs, including erlotinib, revealed sensitive and resistant organoids. The latter were screened for additional alterations and found to be Met-amplified. Consistently, they were sensitive to crizotinib, a Met inhibitor [61].

These studies bring us to one important consideration: the setting up of this organoid cultures and subsequent drug screenings might take up to few months, which is a relatively long time for advancing new therapeutic strategies for late-stage diseases.

## 4. Microfluidic-Based Systems and the Organ-on-a-Chip Technology

To bridge the gap between in vitro and in vivo drug testing, accurate models of tissue physiology are needed. Before a drug can enter a clinical trial, its efficacy and toxicity as well as its absorption-distribution-metabolism-excretion (ADME) profile and pharmacokinetics/pharmacodynamics have to be accurately assessed. Mouse models are widely employed to evaluate these parameters before moving to superior animal models and eventually to the human. Although mouse research will be still fundamental in the years to come, we believe that new approaches are mandatory to overcome the difficulties in handling those models, the costs and the imperfect readouts due non-comparable metabolisms, organ physiology, and immune systems [62,63].

### 4.1. Spheroids and Organoids Cultured in Microfluidic Systems

3D cell cultures such as spheroids and organoids integrated with microfluidic chips have been generated with the purpose of evaluating the efficacy of drugs in a more physiological context. Building up the perfect platform that not only accommodates good amounts of sample but also allows for direct analysis is not an easy task. In 2014, the PDMS Spherochip platform was built by Kwapiszewska and collaborators, consisting in a serpentine inlet for gradient generation and 384 compatible wells for accommodation of the cells. The authors showed that spheroids of two cancer cell lines (Hep-G2 and HT-29) were easily formed in this platform and that it was possible to analyze their proliferative and metabolic responses upon 5-FU administration [64]. As discussed previously, spheroids are an intermediate model of cancer complexity and are suitable for high-throughput screenings. However, a major issue is the lack of size homogeneity in culture, which may result in non-uniform responses as drug penetration can differ widely [65]. Fang and colleagues invented a liquid dome method to generate spheroids with increasing sizes on an agarose chip, which was achieved by simply modulating the surface tension. They showed that spheroids with bigger sizes have a differential response compared to those of small dimensions, presenting their platform as the first to have the ability of producing more than 200 spheroids of different sizes at the same time [66]. Spheroids of uniform size have been prepared in a microfluidic platform for drug testing purposes by Patra and colleagues. They have been able to generate more than 5000 uniform spheroids, which were treated with cisplatin, resveratrol, and/or tirapazamine and evaluated at the single-cell level by flow cytometry. This work confirmed that the initial volume of the spheroid is crucial to achieving reliable pharmacological effects measured as cell proliferation/apoptosis. In addition, the authors observed that drug efficacy does not necessarily correlate with a decrease in spheroid volume, which therefore should not be used as a reference parameter when evaluating drug efficacy [67].

Organoids too can be integrated in microfluidic chips to recapitulate organ structure and functions. A chip with organoids of primary lung cancer has been proposed by Jung and coworkers that allowed for the uniform growth of the organoids, an important characteristic for drug testing purposes. The authors observed that the organoids responded well to cisplatin and etoposide in their outer part while the core showed some degree of insensitivity to chemotherapy [68]. Similarly, Mazzocchi and colleagues have developed a microfluidic culture system by using two patient-derived mesothelioma samples. They showed that such patient-derived organoids respond to common chemotherapeutics in the same way as the patients themselves, and thus confirmed that this system may be used for advancing precision medicine therapeutics [69].

An aspect that we should acknowledge about the use of microfluidic chips is the low sample input necessary to validate the activity of a certain drug. It is indeed true that sample biopsies for research purpose are rare and usually available in small quantities. It has been shown before that sliced tissues retain a certain degree of viability when cultured in vitro [70]. Astolfi and colleagues have implemented a serpentine-shaped chip that can accommodate up to 25 micro-dissected tissue slices trapped by sedimentation. They showed that slices derived both from xenografted cell lines and patient-derived tumors can be maintained in culture for up to 8 days without perfusion and subjected to chemosensitivity assays [71].

As an added value, microfluidic systems represent innovative tools to study the hallmark capabilities of cancer cells. The spheroid culture platform sphero-IMPACT, for example, has the potential to model vasculogenesis and angiogenesis together with tumor cell migration [72]. Nashimoto and colleagues generated a vascularized breast cancer platform and tested anticancer drugs either under perfusion or in static conditions. With this system, they proved that spheroid vascularization, which involves the presence of stromal cells, induced cell proliferation and that under the fluid flow anticancer drugs did not decrease the volume of tumor spheroids [73]. Similarly, Shirure and colleagues designed a microfluidic system to evaluate angiogenesis, tumor growth, and response to therapy. They employed breast cancer organoids and developed a microvascular network where to study the response to both chemotherapeutics and anti-angiogenic drugs [74].

### 4.2. Organ-on-a-Chip Platforms

**Figure 3:**
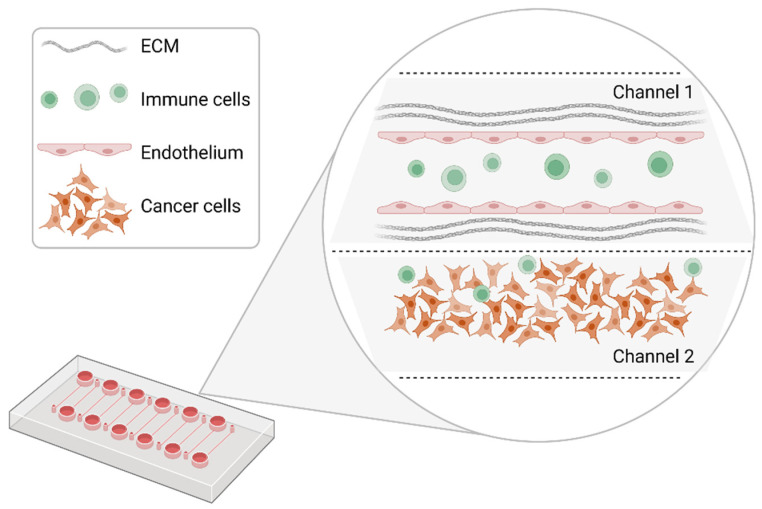
Schematic representation of a model organ-on-a-chip configuration. Within different channels, segregated micropillars of endothelial cells can be cultivated to form a functional vessel. Cancer cells are added and cultivated in the second channel. Immune cells are infused to evaluate their interaction with cancer cells and endothelial cells. Created with BioRender.com.

What distinguishes organ-on-a-chip from the previously described models is the possibility of engineering the tumor microenvironment, so that a more physiological setting is implemented for precision medicine purposes. Microphysiological systems (MPS) based on microfluidic systems have been generated around one decade ago to model cancer complexity. Within these systems, the 3D structure of the tumor, its cellular heterogeneity, extracellular matrix, vasculature, and mechanical stresses (fluid shear forces) can be, at least in part, recreated [75] (Figure 3). MPS, also known as organ-on-a-chip, are miniaturized cell culture platforms made out of polydimethylxiloxane (PDMS) or other clear materials, containing perfusion channels and trap wells to seed cells sometimes embedded in extracellular matrices. These cells recapitulate tissue to organ physiology in higher order model systems, namely the body-on-a-chip technology [76].

Organs-on-a-chip have been only recently exploited to evaluate the potency of drug treatments. Such platforms are still in an early phase of development and the majority of the research so far focuses on demonstrating the reliability of the model rather than using it as a preclinical platform. An attempt to engineer the tumor microenvironment was done by generating a liver-on-a-chip tumor model. Lu and colleagues have proved the feasibility of decellularizing the liver matrix and resuspending the lyophilized content to obtain an extracellular matrix (ECM) suspension to be mixed with GelMA (Gelatin Methacryloyl), a hydrogel used for 3D scaffolds. This will help not only to simulate the mechanical properties of the ECM in vivo, but also to favor cell viability owing to the liver growth factors still contained in it. The group also provided evidence of the usability of this chip for drug toxicity studies [77].

The vascular network is of fundamental importance for nutrient supply and drug distribution within a certain tissue. Vascularized microtissues (VMTs) have been generated on chip by Phan and colleagues. They created a 96-well plate format chip with 12 inlets for multiple drug testing. Within the main tissue chambers, they seeded HCT116 colorectal cancer cells and endothelial cells along with stromal cells to recapitulate a VMT. The process of vasculogenesis, under controlled pressure, took up around 7 days after which a panel of FDA-approved drugs were administered at 1 µM. Some drugs affected both the vasculature and the cancer cells, while some other just the cancer cells. Strikingly, when the same drugs were administered to 2D cell cultures, they showed a completely different activity. For example, mitomycin C, vorinostat, and gemcitabine exerted an antiproliferative effect in VMT and no effect in 2D [78].

With a similar approach, tumor-mimetic chips have been designed by Pradhan and coworkers where vasculogenesis of human breast tumor-associated endothelial cells was established on fibronectin-coated microfluidic channels, followed by the introduction of a mixture of foreskin fibroblasts and either non-metastatic MCF7 or metastatic MDA-MB-231 breast cancer cells embedded in a fibrinogen hydrogel matrix. The chip allowed the growth and monitoring of the cells for 28 days, during which areas of dormant cells and areas of proliferating cells were observed. The chip was either built with a high perfusion (HPC) or a low perfusion (LPC) design to better mimic the irregular morphology of the tumor vasculature in vivo. Such design allowed the visualization of perfusion gradients within the chip by a TRITC-dextran fluorescent marker, and evidence was reported for cell inactivity in areas of poor perfusion. Consistent with this evidence, the authors demonstrated that doxorubicin and paclitaxel treatments were causing tumor shrinkage specifically in the HPC setting, whereas in the LPC only doxorubicin had an appreciable effect [79]. A more recent paper by Cui and colleagues shows that it is in fact possible to reconstitute a glioblastoma tumor niche in a chip made of concentric channels separated by regularly interspaced micropillars so that all the compartments are interacting with each other. In the outer ring they seeded human brain microvascular endothelial cells to pattern 3D brain vessels, in the middle ring human tumor-associated macrophages (TAMs) and patient-derived, molecularly distinct glioblastoma cells in a hyaluronan-rich matrix, while in the central ring they would make space for media supply. This work showed that it is possible to follow the interaction between microvessel-infused allogenic CD8 T-cells and tumor cells as well as TAMs. Mesenchymal glioblastoma showed to have the most immunosuppressive environment, with less CD8 T-cells and more TAMs that were recruited by tumor cells. Moreover, the authors demonstrated that the combined inhibition of colony-stimulating factor 1-receptor (CSF-1R) and programmed death 1 (PD-1) reverted this phenotype [80].

Finally, an area under intense investigation is the use of lab-on-chips technologies to model biological barriers. Cell-based models of intestinal, lung, and blood-brain barrier are presented by Walter and colleagues. They employed a PDMS platform together with measuring electrodes to define the trans-epithelial-electrical resistance (TEER) within the epithelial layer of intestinal Caco2 cells, lung A549 cells, and human hCMEC/D3 brain endothelial cells. They also modeled the blood-brain-barrier (BBB) by coculturing brain endothelial cells together with pericytes and glial cells showing that brain endothelial cells in this BBB model display a higher TEER and lower permeability to sodium fluorescein, isothiocyanate-labeled- dextran and blue-labeled albumin. The chip was design to assess epithelial permeability under no or low flow by microscopic visualization [81].

The organ-on-a-chip technology, despite being still at the proof-of-concept stage, shows encouraging results. From the culture of microtissues to the recreation in vitro of vascularized tissues, it is now possible to reconstitute the tumorigenic environment in vitro and to cultivate cancer cells for long periods of time owing to the possibility of removing metabolic cell waste by application of a fluid flow. A low cell input is convenient for studies where a little amount of material is available and the low input medium makes it feasible to test a large number of drugs at a low cost. Moreover, it is worth mentioning that although being far from reconstituting an entire body-on-a-chip, these models can be used in toxicity studies with reliable results. One example for all is the attempt of Skardal and colleagues: they reconstituted a 6-tissues body-on-a-chip by seeding organoids of liver, heart, vasculature, testis, and either colon or brain in a 96-well plate and on different matrices. The platform was assembled with a recirculation of fluids, so that pro-drugs such as capecitabine could be administered and processed by the liver organoids, and toxicity evaluated on downstream organoids. This elegant study also demonstrated that toxicity was not seen in downstream organoids when liver metabolism was bypassed [82].

## 5. Conclusions

It has been our intention to provide the reader with an overview of how the advanced cellular models are becoming more and more common platforms to conduct drug screenings in vitro.

We foresee that animal research on genetically modified mice and patient-derived xenografts will be partially replaced by in vitro studies. It is in fact true that, despite being really informative, this kind of research is costly and labor-intensive. Furthermore, establishment of these models requires time (months), which often the patient does not have, and expertise. It is difficult, if not impossible, to achieve high-throughput results in animal models, whereas it is totally feasible in vitro with spheroids and organoids. The latter are going through a phase of standardization that involves the selection of uniform sizes for more reliable drug-response results. The bioptic sample required for establishing these models is minimal and expansion can be done in weeks, after which drug compounds can be tested. The final goal would be to set up patient-derived models in a time frame that would allow for testing drugs or combination of drugs before administering them to the patient. In the worst scenario, to at least exclude the ones that do not work. To this aim, the fidelity of the selected model is of extreme importance: the introduction of elements of cancer complexity is a priority for result reliability.

If spheroids are aggregates of cells and stemness models that grow in suspension, so not in a “stiff” matrix, organoids include this aspect and are a real reflection of the tumor architecture. However, not all cancers have yet a collectively accepted representation in terms of 3D models: we are referring to the hematological malignancies for which long-term cultures that preserve the initial tumor heterogeneity are not available.

Microfluidics and organ-on-a-chip technologies aid in introducing more elements of the tumor microenvironment: stromal, vascular, and immune cells, as well as the ECM. This approach produces more comprehensive readouts, as the microenvironment plays a central role in regulating the response to therapy. We have described models that, in the time-frame of weeks and with a low-input material, build up a tissue on a chip. In such models, cells can be cultured for up to a month owing to the fluid flow that removes cell waste. The overall picture is that of tightly controlled, multicellular systems to investigate drug efficacy and toxicity.
